# The Swansea Hospital Dispute

**Published:** 1898-10-22

**Authors:** 


### THE SWANSEA HOSPITAL DISPUTE.

We have carefully gone into the whole matter of the dispute which has lately been in progress at the Swansea Hospital in regard to the temporary use of the ophthalmic out-patient room by the ordinary patients on the days when it is not required for the ophthalmic out-patients, and we cannot but feel that the Hospital Committee has made a false step, and set up a very awkward precedent in giving way as they have done to the very high-handed proceedings of Dr. Jabez Thomas. Nothing could exceed the courtesy of the chairman, Mr. Howel Watkins, in his efforts on two successive occasions to make it easy for Dr. Thomas to reconsider the position he had taken up, a position which, when one considers that in essence it hinges on an assumed difference between ordinary and ophthalmic out-patients as carriers of septic material, savours somewhat of the absurd. Dr. Davidson founded his objection on legal matters, but Dr. Jabez Thomas took quite a different line, and maintained that in turning out the furniture which had been placed in the room by the order of the board, and fumigating the rooms just when they were required for the out-patients, he acted purely in the interests of his patients, and said that if similar furniture were brought there again he was afraid he would have to act again in the same manner. So far as this is a matter of asepticity and cleanliness, if the out-patient room is so near the operating-room as to make its use by one sort of out-patient unsafe, we can hardly look upon it as safe for use by out-patients of another kind. In any case his action is a positive and not altogether dignified revolt against the orders of those responsible for the management of the institution, and we think that the committee somewhat overstepped the proper limits of meekness in accepting his action as they did. Many a hospital surgeon may, perhaps, wish that he could ride rough-shod in such a manner over his committee; but such despotic proceedings are not good for institutions. It is difficult to maintain discipline if it is not maintained equally all round; and one must not forget that there is more than one surgeon to the Swansea Hospital. If they were all to retaliate in the same manner as Dr. Jabez Thomas seems to have done, whenever the House Committee takes any action which is displeasing to them, the patients would suffer greatly, and the place would become a pandemonium.

